# Treatment of Rheumatism

**Published:** 1892-08

**Authors:** Chas. W. Brown


### TREATMENT OF RHEUMATISM.

BY CHAS. W. BROWN, M. D.

October 20, 1891, saw Mrs. H., of this city, aged 44 years. Found her suffering from rheumatism; there was pain, redness and swelling in both knees and ankles. Temperature 103, skin hot and dry, considerable thirst; urine scanty and high colored. She said she had been subject to such attacks for several years, whenever exposed to wet and cold weather, and the duration of the attacks was from three to six weeks; and she had been confined to her bed for several weeks at a time. For five days previous to my first visit she had been treated by salicylate of sodium, aconite and opium, without any perceptible benefit. I prescribed W. R. Warner's Elixir Salicylic Acid Comp., a dessertspoonful every four hours, and gradually increased the dose to a tablespoonful. In less than twenty-four hours she was perspiring freely, the temperature fell to 99, and she was free from pain; the urine became more abundant, and lighter in color, the swelling of the joints rapidly disappeared, and in one week she could walk without difficulty. She continued to take the elixir for two weeks longer, in dessertspoonful doses, three times a day, and has had no return of the disease up to this date.*

Case 2—Mrs. G. W. D., aged 28 years, Elmira, N. Y., October 11, 1889. Had acute rheumatism; swelling and redness of knees, ankles and feet, also left hand and arm perfectly helpless; pain severe and constant; temperature at first visit 104½, pulse rapid and irregular, occasional sharp pains through left breast. Prescribed Warner's Elixir Salicylic Acid Comp., tablespoonful every four hours, quinia sulph., gr. ij, every four hours. Fomented limbs by means of

*Elixir Salicylic Acid Comp. is an elegant preparation, composed as follows: Each tablespoonful contains 24 grs. Salicylate Sodium, so combined with cimicifugæ, potass. iodide and gelseminum as to avoid all unpleasant results, such as gastric and intestinal irritation, nausea, delirium, restlessness and rapid breathing, which so often follows the administration of salicylic acid.

The dose is from a tablespoonful to a teaspoonful, as the case may require. Prepared by Wm. R. Warner & Co., Manufacturing Chemists, Philadelphia and New York.

flannel cloths wrung out of hot salt water. She improved from commencement of treatment, and walked out on the street ten days later, and has had no return of the disease.

Case 3—G. W., locomotive fireman, aged 25 years. Was called to treat him March 22, 1888. He has been unable to leave his bed for two weeks; ankles and feet were enormously swollen and red, very tender to touch, and painful. So he was obliged to take full doses of opium to obtain sleep. Prescribed Warner's Elix. Salicylic Acid Comp., f oz. vj; Potas. iodidi, dr. iij. M. Sig.—Tablespoonful every six hours. Fomentations as in former case. In a week he was able to walk about, and two weeks from beginning of treatment he went to his usual work as fireman on railroad. This man had gonorrhœa, when swelling of ankles came on after he had his clothing wet through by being out in a storm of sleet and rain, and the discharge suddenly stopped, and did not return until profuse perspiration took place as a result of treatment and reduction of temperature. This discharge continued for ten days after he commenced work, but finally yielded to appropriate treatment.

Case 4—J. O. D., woman, aged 45 years. Chronic rheumatism, right leg and hip; also both hands. Been helpless for eight months. Has tried various plans of treatment without material benefit. Ordered tablespoonful of the elixir four times a day (added to each twelve-ounce bottle, dr. iij., potas. iodidi); also gave 2 gr. pill quinia sulph. three times a day. She gradually improved, and in four weeks was able to go up and down stairs without difficulty, though the enlargement of joints remained for several months. She continued to take the elixir for four months, and has had no return of the disease sufficient to prevent her from being around and being able to perform her usual household duties. She has had, occasionally, symptoms of a return of the lameness, but has been relieved by taking the remedy for a few days.

*902 Fourteenth St., N. W., Washington, D. C., December 14.*

N. B. Wm. R Warner & Co. promise to send some of this elixir for gratuitous use to any physician who has an obstinate case of rheumatism to treat.—*Southern Clinic.*

